# LGBTQ+ Aging in Precarious Times: Reimagining Horizons From Radical Margins

**DOI:** 10.1093/geroni/igaf122.1069

**Published:** 2025-12-31

**Authors:** Austin Oswald, Karen Fredriksen-Goldsen

## Abstract

LGBTQ+ older adults live within a dynamic sociopolitical context in which their access to human rights and freedoms across the life course is tenuous and fraught. Their experiences of political oppression are embedded in a long history of legal and social change, demonstrating the deleterious effects of anti-LGBTQ+ legislation on health while also highlighting community-based approaches to resistance. This symposium illuminates the intersectional lives of LGBTQ+ older adults to identify innovative strategies that foster resilience in the face of adversity. The first paper, presented by Dr. Oswald, employs participatory action research to illustrate how leisure can serve as both a site of oppression and a tool for resistance, demonstrating how culturally responsive leisure activities promote healthy aging among older Black lesbians. The second paper, by Dr. Prasad, utilizes statistical methods to critically examine how interpersonal network ties and environmental policy contexts shape transgender older adults’ social support resources. The third paper, by Dr. Jen, applies creative research methods to analyze life course interviews with older bisexual individuals, poetically narrating their life stories including themes of struggle, survival, and healing. These papers center the perspectives of overlooked subgroups within the LGBTQ+ community—older Black lesbian, transgender, and bisexual adults—to explore how their aging experiences both shape and are shaped by the sociopolitical context. Together, they envision new horizons in gerontology by drawing on the wisdom of those living at the margins of society, offering a critical lens to reimage health and aging.

